# A cross-sectional evaluation of HIV testing practices among women in the rural Dominican Republic

**DOI:** 10.1186/s12905-020-0891-2

**Published:** 2020-02-07

**Authors:** Madeline C. Montgomery, Zachary Alholm, Alexi Almonte, Kevin J. Sykes, Gregory Rudolph, Brandon Cusick, Laura Castello, Genoviva Sowemimo-Coker, Irene Tang, Sarah Haberlack, Philip A. Chan

**Affiliations:** 1 Department of Medicine, Brown University, Providence, RI USA; 2 University of Kansas Medical Center, Kansas City, Kansas USA; 3 World Outreach Foundation, Kansas City, MO USA

**Keywords:** HIV, STIs, Dominican Republic, HIV testing, Women

## Abstract

**Background:**

The Dominican Republic (DR) ranks among nations with the highest burden of HIV in the Caribbean. Cultural and gender roles in rural areas of the DR may place women at increased HIV risk. However, little is known about sexual health and HIV testing behaviors among women in the rural DR.

**Methods:**

We conducted a needs assessment among a systematic sample of adult women in a rural DR community in 2016. Demographic and behavioral attributes related to HIV testing, sexual health, and healthcare utilization were evaluated. Poisson regression analysis was used to identify demographics and behaviors associated with having had a previous HIV test. Significance was defined as a *p*-value < 0.05.

**Results:**

Among 105 women evaluated, 77% knew someone with HIV and 73% of women reported that they would be very or extremely likely to take an HIV test if offered. Only 68% reported a previous HIV test, including 47% who were tested over 2 years prior. Barriers to HIV testing included low risk perception (23%), distance or requisite travel (13%), and discomfort being tested (11%). Women who had never been tested for HIV were more likely than those who had been tested to be older (*p* = 0.03), to have a lower level of education (*p* = 0.04), and to have never been tested for other sexually transmitted infections (STI; *p* <  0.01). In the Poisson multiple regression model, the only significant predictor of having had an HIV test was having had an STI test (*p* = 0.03).

**Conclusions:**

In the rural DR, numerous barriers contribute to low prevalence of HIV testing among women. Most women report willingness to have an HIV test and many engage in routine health care, indicating that this population may benefit from incorporating HIV testing and other sexual health promotion activities into routine medical care.

## Background

The HIV epidemic continues to be a significant cause of morbidity and mortality worldwide [1–3]. The Caribbean has the second highest rate of HIV infection in the world after sub-Saharan Africa. In the Caribbean, 75% of people with HIV reside in either the Dominican Republic (DR) or Haiti [4], and over half of adults living with HIV are women [5]. Although HIV rates in the DR have decreased, with at least 50% lower HIV incidence in 2012 compared to 2001 [5], HIV remains a critical public health concern in the region.

HIV prevalence among the general population in the DR is estimated to be 1% [5]. In the capital city of Santo Domingo, HIV prevalence among those tested declined from its peak of 2% in 1995 to 1.1% in 1999, where it has stabilized through 2006, the last year with available data [6]. In general, significant disparities still exist among different groups with regard to rates of HIV infection [5, 6]. Women ages 20–24 living in the DR are almost twice as likely as men in that age group to be infected with HIV [7], and female sex workers have an HIV prevalence of 1–4% [6]. Gay, bisexual, and other men who have sex with men (MSM) are also a high-risk group, accounting for 33% of new HIV infections annually in the DR [5]. Additionally, limited studies throughout other Caribbean countries have demonstrated high HIV prevalence is attributable to female sex work [8]. Risk of HIV infection is also disproportionate across ethnic groups. Haitians living in the DR display significantly greater HIV incidence than non-Haitians [9], likely due in part to language barriers, lack of education [4], poverty, and social disruption related to migration [10].

Most estimates of HIV prevalence in the DR are based on studies in larger urban centers. However, HIV prevalence may differ across geographic setting. One study in *bateyes*, Dominican sugar farms on which many women of Haitian ancestry work, found high rates of HIV infection comparable to those among female sex workers in the DR [10]. Otherwise, information on rural HIV prevalence in the Caribbean is scarce. Similarly, HIV testing and risk behaviors associated with infection have predominantly been studied in urban areas [11]. Behaviors and access to sexual healthcare including HIV testing in rural populations are largely unknown. A Jamaican study found that adolescents in remote areas are more likely to be uninformed or misinformed about HIV than their urban counterparts, and that girls in rural Jamaica have a lower HIV risk perception than those in urban areas despite engaging in high-risk behaviors [12]. This knowledge gap may contribute to disparate HIV rates within other Caribbean countries, including the DR. Studying HIV prevalence and attitudes in rural populations is crucial in order to understand and address the HIV epidemic in the Caribbean as a whole.

Improving HIV testing and subsequent linkage to care are critical components in addressing the HIV epidemic in the DR. However, minimal data are available on these efforts, especially in the rural DR. Significant barriers to HIV testing, access to treatment, and retention in care have been described in other Hispanic/Latino countries. These include stigma [13–16], transportation, cost [14], low risk perception, lack of education [12], and other individual- and structural-level barriers [13, 15, 17]. For example, some HIV patients describe choosing clinics further from their communities in order to avoid stigma, which increases the transportation time and related costs they face [14]. These factors may lead to delayed HIV diagnoses and increased complications from HIV/AIDS [18]. Identifying barriers to HIV testing and care is the first step in the design and development of effective public health interventions.

The goal of the present study was to review the results of a community-based needs assessment performed in 2016 of sexual and reproductive health that was performed by public health workers in Constanza, DR. Constanza is a rural agricultural community of about 60,000 people located in the central part of the country. In the DR, as in other Caribbean countries, the majority of new HIV cases occur due to heterosexual contact [19]. Women are especially vulnerable because cultured gender roles may subject women to power imbalances in sexual decision-making [20]. In general, male-dominated decision-making can impede the ability for women to negotiate condom use, thus increasing their risk of obtaining HIV from their infected male partners [21]. By addressing the sociocultural barriers women face such as structural sexism [4], lack of independent economic opportunity [10], and lower ability to negotiate condom use [10, 20], efforts to empower women can reduce their risk of becoming infected with HIV [22, 23]. The results of this sexual and reproductive health needs assessment among women in the rural DR will help inform future health promotion initiatives.

## Methods

### Setting

In May 2016, the World Outreach Foundation (Kansas City, Missouri), in collaboration with the United States Peace Corps, conducted medical clinics in Constanza, DR. In order to inform services delivered through the clinics, staff from these organizations conducted an in-depth needs assessment of the surrounding area. Key informant interviews indicated that one specific community had a lower socioeconomic status and a high suspected prevalence of HIV compared to other areas. This community of several thousand residents was anecdotally reported to include women who engaged in transactional sex. As there were no census or other surveillance data available, demographics and behavioral characteristics of residents were largely unknown. The needs assessment focused on women due to previously documented gender-based disparities in healthcare access [24], economic agency [10], and medical and reproductive health sequelae of HIV, other sexually transmitted infections (STIs), and pregnancy [25–27] in the Caribbean.

#### Needs assessment

A needs assessment of this community was performed in 2016 which collected information on demographics, healthcare access, obstetric and gynecological health, contraception use and knowledge, HIV knowledge and testing history, sexual history, and acceptability of HIV testing. Spanish-speaking program staff verbally administered the one-time needs assessment survey in person at individual homes, with each assessment taking approximately 15–20 min to complete. Questions were based on previous survey measures used for public health purposes [28] Given the project goal of evaluating the need for HIV prevention services in this community, the primary area of interest was history of, and future amenability to, HIV testing. Staff focused efforts on a target sample size of 100 individuals. The target sample size *n* = 100 was calculated based on a conservative theoretical proportion of 50% being amenable to HIV testing and allowing for a 10% margin of error, yielding a 95% confidence level to detect differences with a *p*-value of < 0.05.

To maximize diversity in the sample, program staff followed a systematic random sampling approach to conduct the needs assessment. All houses in the community were counted (*n* = 270) and numbered sequentially (i.e., from 1 to 270). To determine which houses to approach for the needs assessment, the number of houses in the community (*n* = 270) was divided by the target sample size (*n* = 100), resulting in the sampling interval *k* = 2. Program staff selected a random starting house and proceeded to sample from every *k*th, or every second, house. Staff attempted to survey one woman of reproductive age (age 18–49) in each household. If no one meeting assessment criteria was available, staff attempted three total visits. If more than one woman of reproductive age lived in the house, staff selected the woman nearest to the head of household.

### Data analyses

The primary use of needs assessment data was informing subsequent delivery of HIV prevention services in the community. In this retrospective analysis of needs assessment data, we aimed to describe HIV testing practices across demographic, social, and behavioral characteristics. We calculated frequencies for each demographic and behavioral variable and tested the distribution of variables across groups using Fisher’s exact tests. Bivariate and multivariable Poisson regression analyses with robust standard errors was used to determine correlates of lifetime history of HIV testing. Relative risk, the output of Poisson regression, more accurately approximates risk compared to the odds ratio for outcomes with greater than 10% prevalence [29]. The robust standard error adjusts for overestimated variance in the case of a binary dependent variable in a Poisson model [30]. Model covariates were selected from among needs assessment variables based on determinants of healthcare access identified in prior studies and were tested for collinearity prior to inclusion in the final model. Significance was defined at two-tailed *α* = 0.05. All statistical analyses were performed in Stata/SE 13.1 [31].

Retrospective review of de-identified needs assessment data was approved by The Miriam Hospital Institutional Review Board (IRB). Data were stored using Research Electronic Data Capture (REDCap), a HIPAA-compliant data management system [32].

## Results

### Demographics and behaviors

A total of 105 women were surveyed. Twenty-three percent were between the ages of 16 and 29 years old, 33% were between 30 and 49 years old, and 44% of women were 50 years old or older (Table 1). Seventy-nine percent had lived in the community for more than 10 years, and 68% had an education level of primary school or less. Sexual debut at 15 years of age or younger was reported by 51% of women surveyed, yet a larger majority (88%) had two or fewer sex partners in their lifetime. Seventy-seven percent of the surveyed women reported knowing someone else with HIV. Although 73% of women reported that they were very or extremely likely to be willing to take an HIV test, fewer (68%) reported having had a previous HIV test. Only 13% of women reported that they were unlikely to be willing to take an HIV test.

**Table 1:** Demographic and behavioral characteristics of adult women in rural Constanza, Dominican Republic, by HIV testing history

	No prior HIV test (ever/unsure)	Prior HIV test (ever)	Total	Fisher’s exact test *p*-value
	*n* = 34	*n* = 71	*n* = 105	
	*n (%)*	*n (%)*	*n (%)*	
Age (years)				
16 to 29	5 (14.7)	19 (26.8)	24 (22.9)	**0.027**
30 to 39	3 (8.8)	17 (23.9)	20 (19.1)	
40 to 49	4 (11.8)	11 (15.5)	15 (14.3)	
50+	22 (64.7)	24 (33.8)	46 (43.8)	
Time lived in the community (years)				
10 or fewer	6 (17.7)	16 (22.5)	22 (21.0)	0.381
More than 10	28 (82.4)	55 (77.5)	83 (79.1)	
Education level				
Less than primary	17 (50.0)	16 (22.5)	33 (31.4)	**0.038**
Primary	10 (29.4)	26 (36.6)	36 (34.3)	
Some high school	4 (11.8)	13 (18.3)	17 (16.2)	
High school or higher	3 (8.8)	16 (22.5)	19 (18.1)	
Age at sexual debut				
15 younger	21 (61.8)	33 (46.5)	54 (51.4)	0.306
Older than 15	12 (35.3)	32 (45.1)	44 (41.9)	
Don’t know/prefer not to answer	1 (2.9)	6 (8.5)	7 (6.7)	
Number of lifetime sex partners				
1	10 (29.4)	21 (29.6)	31 (29.5)	0.708
2 or more	23 (67.7)	43 (60.6)	66 (62.9)	
Don’t know/prefer not to answer	1 (2.9)	7 (9.9)	8 (7.6)	
Pregnant, ever				
No	3 (8.8)	5 (7.0)	8 (7.6)	0.712
Yes	31 (91.2)	66 (93.0)	97 (92.4)	
Received STI test, ever				
No/unsure	32 (94.1)	44 (62.0)	76 (72.4)	**< 0.001**
Yes	2 (5.9)	27 (38.0)	29 (27.6)	
Know someone with HIV				
No	9 (26.5)	15 (21.1)	24 (22.9)	0.621
Yes	25 (73.5)	56 (78.9)	81 (77.1)	
Likelihood of taking HIV test if offered				
Very or extremely likely	24 (70.6)	53 (74.7)	77 (73.3)	0.847
Somewhat likely	3 (8.8)	7 (9.9)	10 (9.5)	
Very or extremely unlikely	6 (17.7)	8 (11.3)	14 (13.3)	
Don’t know/prefer not to answer	1 (2.9)	3 (4.2)	4 (3.8)	

Bold numbers are significant

### HIV testing

Women who had never been tested for HIV (*n* = 34) were more likely than those who had been tested (*n* = 71) to be older (*p* = 0.03), to have a lower level of education (*p* = 0.04), and to have never been tested for STIs (*p* <  0.01; Table 1). Among women who had never been tested, the most commonly endorsed barriers to HIV testing access (non-mutually exclusive) were lack of perception of risk (23%), distance to a testing site (13%), and feeling uncomfortable seeking testing (11%). Age of sexual debut, number of sexual partners, knowing someone with HIV, education level, and willingness to be tested did not significantly differ between women who had and had not ever had an HIV test. When adjusted for age, education level was also not significantly associated with HIV testing. In the Poisson multiple regression model adjusting for demographic and behavioral factors, the only significant predictor of having ever had an HIV test was having ever had an STI test (*p* = 0.033, Table 2).

**Table 2:** Incident relative risk (IRR) of no past HIV test among women in rural Constanza, Dominican Republic, unadjusted and adjusted (aIRR) for demographic and behavioral indicators

	IRR	95% CI	aIRR	95% CI
Age (years)				
16 to 29 years	1.00	–	1.00	–
30 to 39 years	0.720	(0.195, 2.67)	0.694	(0.172, 2.80)
40 to 49 years	1.280	(0.405, 4.05)	1.22	(0.314, 4.76)
50+ years	2.300	(0.991, 5.32)	1.73	(0.581, 5.17)
Time lived in the community (years)				
10 years or less	1.00	–	1.00	–
More than 10 years	1.240	(0.137, 0.541)	0.627	(0.211, 1.86)
Education level				
Less than primary	1.00	–	1.00	–
Primary	0.539	(0.289, 1.01)	0.649	(0.338, 1.25)
Some high school	0.457	(0.181, 1.15)	0.621	(0.200, 1.93)
High school or higher	0.307	(0.102, 0.916)	0.700	(0.216, 2.27)
Age at sexual debut				
15 years or younger	1.00	–	1.00	–
Over 15 years	0.701	(0.389, 1.26)	0.782	(0.431, 1.42)
Unknown/prefer not to answer	0.367	(0.058, 2.35)	0.502	(0.099, 2.55)
Number of lifetime sex partners				
1	1.00	–	1.00	–
2 or more	1.080	(0.587, 1.99)	1.120	(0.636, 1.96)
Unknown/prefer not to answer	0.388	(0.057, 2.62)	0.454	(0.052, 3.96)
Pregnant, ever				
No	1.00	–	1.00	–
Yes	0.852	(0.331, 2.19)	0.751	(0.280, 2.01)
Received STD test, ever				
No/unsure	1.00	–	1.00	–
Yes	0.164	(0.323, 0.549)	0.219	(0.054, 0.887)
Know someone with HIV				
No	1.00	–	1.00	–
Yes	0.823	(0.446, 1.52)	0.876	(0.495, 1.55)
Likelihood of taking HIV test if offered				
Extremely or very unlikely	1.00	–	1.00	–
Somewhat likely	0.700	(0.226, 2.16)	0.784	(0.307, 2.00)
Extremely or very likely	0.727	(0.364, 1.45)	1.070	(0.554, 2.05)
Unsure	0.583	(0.095, 3.57)	0.669	(0.138, 3.25)

*CI* Confidence interval

Among those who had received an HIV test (*n* = 71), 45% had their most recent test less than a year ago, 18% within one to 2 years, and 37% more than 2 years ago (Table 3). Most (65%) were tested at a hospital, with only 6% reporting being tested at the public health clinic located in the community. Fifty-nine percent of women reported having no difficulty accessing HIV testing. For those who reported facing challenges (*n* = 43), the biggest barriers to accessing HIV testing included low risk perception (56%), distance (33%), and not feeling comfortable being tested (26%). Among those who were unwilling to take an HIV test (*n* = 34), the biggest reasons included stigma (9%) and low HIV risk perception (9%). However, 38% of women did not feel comfortable answering this question.

**Table 3:** HIV testing access and utilization among adult women in rural Constanza, Dominican Republic

	% (n)
Most recent HIV test, among ever tested (*n* = 71)	
< 1 year ago	45.1 (32)
1–2 years ago	18.3 (13)
> 2 years ago	36.6 (26)
Location of most recent HIV test, among ever tested (*n* = 71)	
Hospital	64.8 (46)
Private clinic	15.5 (11)
Public clinic in community	5.6 (4)
Public clinic outside community	1.4 (1)
Private residence	1.4 (1)
Difficulty accessing HIV test	
Cost of test	6.7 (7)
Cost of transportation	6.7 (7)
Access to transportation	6.7 (7)
Distance	13.3 (14)
Don’t know where to access	1.0 (1)
Husband will not allow	1.0 (1)
Do not feel comfortable accessing	10.5 (11)
Stigma	6.7 (7)
No perceived risk	22.9 (24)
Other	1.9 (2)
No difficulty indicated	59.0 (62)
Reasons for unwillingness to take HIV test, among never tested (*n* = 34)	
Fear of positive result	5.9 (2)
Prefer not to know status	0.0 (0)
Stigma	8.8 (3)
No perceived risk	8.8 (3)
Believe HIV diagnosis does not make a difference	0.0 (0)
Religion	0.0 (0)
Fear tests are used multiple times	2.9 (1)
No reason indicated	35.3 (12)
Prefer not to answer	38.2 (13)
Reasons for unwillingness to take HIV test, among unlikely to test (*n* = 14)	
Fear of positive result	0.0 (0)
Prefer not to know status	7.1 (1)
Stigma	7.1 (1)
No perceived risk	50.0 (7)
Believe HIV diagnosis does not make a difference	7.1 (1)
Religion	7.1 (1)
Prefer not to answer	21.4 (3)

### Access to contraception

We also explored access to, and use of, contraception among women in the cohort (*n* = 105, Table 4). Seventy-six percent of women knew where to access contraception if needed. Locations included the local hospital (35%), the local public clinic (21%), or other public clinics (13%). Ninety-five percent of women had heard of male condoms. Fewer women had heard of oral contraceptive pills (79%) and intrauterine devices (IUDs; 50%, Table 4). Out of the total (*n* = 105) women surveyed, 56% (*n* = 59) had ever used contraception, with the most common types being oral contraceptive pills (66%) and male condoms (30%). In total, 16% of women reported difficulty accessing contraception services with cost and access being the most common reasons.

**Table 4:** Contraceptive knowledge and utilization among women in rural Constanza, Dominican Republic

	% (n)
Know where to access contraception	
Yes	76.2 (80)
No	23.8 (25)
Where would go to access contraception	
Hospital	35.2 (37)
Community clinic	21.0 (22)
Other public clinic	13.3 (14)
Private clinic	1.9 (2)
Pharmacy	4.8 (5)
Not applicable	23.8 (25)
Contraception methods ever heard of^a^	
Tubal ligation	68.6 (72)
Vasectomy	46.7 (49)
Intrauterine device	49.5 (52)
Injectables	72.4 (76)
Hormonal implant	65.7 (69)
Pill	79.0 (83)
Male condom	95.2 (100)
Female condom	50.5 (53)
Emergency contraception pill	58.1 (61)
Rhythm method/fertility awareness	54.3 (57)
Lactational amenorrhea	57.1 (60)
Withdrawal	78.1 (82)
Other	3.8 (4)
Contraception use, ever	
Yes	56.2 (59)
No	43.8 (46)
Types of contraception used^a^	
Condom	17.1 (18)
Birth control pill	37.1 (39)
Tubal ligation	3.8 (4)
Injectables	8.6 (9)
Implant	1.0 (1)
Emergency contraception pill	1.9 (2)
Lactational amenorrhea	1.0 (1)
Withdrawal	2.9 (3)
Difficulty accessing contraception services	
Yes	16.2 (17)
No	83.8 (88)
Barriers to contraception access^a^	
Cost of healthcare	7.6 (8)
Cost of transportation	5.7 (6)
Access to transportation	3.8 (4)
Distance	6.7 (7)
Don’t know where to go	1.9 (2)
Husband will not allow	1.0 (1)
Other family member will not allow	1.9 (2)
Do not feel comfortable accessing	5.7 (6)
Stigma	5.7 (6)
Other	9.5 (10)
No barriers indicated	70.5 (74)

^a^These response options were not mutually exclusive; response distribution may exceed 100%

### Access to health care

We also explored access to general health care among women in the cohort (*n* = 105, Table 5). Seventy-five percent reported visiting a medical provider twice a year. Eighty-five percent of women had a medical visit in the last year. The last medical visit was most commonly reported to have taken place at a hospital (50%), followed by the local public health clinic (20%). Forty-seven percent reported difficulty accessing healthcare services. Barriers included cost (33%), distance (18%), cost of transportation (14%) feeling uncomfortable going (9%) and stigma (8%). Sixty-nine percent of women reported having ever had a pap smear before. Only 29% reported a pap smear in the last year.

**Table 5:** General healthcare access and utilization among adult women in rural Constanza, Dominican Republic

Characteristic	% (n)
Frequency of medical visits	
Twice per year	75.2 (79)
Once per year	17.1 (18)
Once every 1–2 years	3.8 (4)
Less than every 2 years	2.9 (3)
Never	1.0 (1)
Last medical visit	
Within past year	84.8 (89)
1–2 years ago	8.6 (9)
More than 2 years ago	3.8 (4)
Never/unsure	2.9 (3)
Location of last medical visit	
Hospital	49.5 (52)
Private clinic	12.4 (13)
Public clinic in community	20.0 (21)
Public clinic outside community	14.3 (15)
Other	3.8 (4)
Difficulty accessing healthcare, ever	
Yes	46.7 (49)
No	53.3 (56)
Barriers to healthcare access	
Cost of healthcare	33.3 (35)
Cost of transportation	14.3 (15)
Access to transportation	9.5 (10)
Distance	18.1 (19)
Don’t know where to access	1.9 (2)
Stigma	7.6 (8)
Feel uncomfortable accessing	8.6 (9)
Family member will not allow	3.8 (4)
Other	10.5 (11)
No barriers indicated	40.0 (42)
Pregnant, ever	
Yes	92.4 (97)
No	7.6 (8)
Total number of live births	
0	7.6 (8)
1	8.6 (9)
2–3	40.0 (42)
4–5	21.0 (22)
6–10	19.0 (20)
More than 10	3.8 (4)
Location of most recent delivery	
Hospital	80.0 (84)
Outpatient center	4.8 (5)
Someone else’s home	1.0 (1)
Own home	4.8 (5)
Other public clinic	1.9 (2)
Not applicable (no delivery)	7.6 (8)
Prenatal vitamin use during last pregnancy	
Yes	64.8 (68)
No/unknown	27.6 (29)
Not applicable (no pregnancy)	7.6 (8)
Had Pap test, ever	
Yes	68.6 (72)
No	26.7 (28)
Unknown	1.0 (1)
Time of most recent Pap test	
Less than 1 year ago	28.6 (30)
1 to 2 years ago	21.0 (22)
More than 2 years ago	21.9 (23)
Unknown	28.6 (30)

## Discussion

This study is among the first to describe HIV testing and other sexual health behaviors among women in the rural DR. The study revealed suboptimal rates of HIV testing despite suspected high epidemiological risk for acquiring HIV [8]. Seventy percent of the sample reported being likely to take an HIV test if offered, although numerous individual- and structural-level factors posed barriers to HIV testing and accessing other healthcare services. Findings from this study indicate the importance of ongoing research and interventions to promote women’s access to HIV and other sexual health services in the rural DR.

Women in the rural DR experience many risk factors associated with HIV. Although women in this sample generally had a low number of sexual partners, this may be offset by high HIV prevalence in the community or risk behaviors of male partners. One of the primary modes of HIV transmission is condomless heterosexual sex, often related to sex work [9]. Gendered social norms for sexual behavior allow men in the DR to have more sexual partners or engage in other high-risk behaviors, placing women, who face biologically greater HIV risk as the receptive partner, at higher risk of HIV infection [33]. Few studies have examined women’s perceptions of their own HIV risk, but limited data collected in this region demonstrate that women perceive themselves to be at lower risk than their behaviors suggest [33]. Low risk perception was an important individual-level barrier to HIV testing in our study, and future HIV prevention efforts in this population should seek to address risk perception as a means of promoting engagement in HIV testing.

Women in this sample demonstrated low awareness of contraceptive methods. The lack of contraceptive knowledge among women in this community likely contributes to elevated HIV risk as well as other negative sexual health outcomes. Though most forms of contraception were familiar by at least half of the sample, use of any of these contraceptive methods was low. For instance, 95% of women were familiar with male condoms. However, only 17% had ever used them during sex. Alongside cultural and gender roles normalizing multiple sex partners for men, sex work and nonuse of condoms in primary heterosexual relationships [23] may contribute to women’s increased vulnerability to HIV acquisition [4]. Studies in similar populations have demonstrated that women’s empowerment, as measured by education and economic agency, is a protective factor for negotiating condom use in heterosexual relationships [20, 23]. Initiatives to empower women to advocate for safer sexual behaviors may ameliorate the burden of HIV and other STIs in this group.

Despite high HIV prevalence in the rural DR [4], only 68% of women in this community had ever had an HIV test. We did not find a significant correlation between education and HIV testing. We had expected people with higher levels of education to be more likely to get tested for HIV due to the fact that education may increase HIV knowledge and condom use [34]. In other populations, greater education is associated with lower prevalence of HIV [6], more accurate perception of HIV risk, and less stigma [4]. We did, however, find that women who had been tested for STIs were significantly more likely to have been tested for HIV as well. Women who successfully overcame barriers to STI testing likely did the same for HIV. Further study should seek to clarify the factors associated with HIV testing to pinpoint targets for efforts to increase uptake.

In addition to providing evidence for the need for increased HIV research and prevention among women in the rural DR, the purpose of this needs assessment was in part to determine whether women in this community would be open to HIV testing conducted by the project staff in the future. Compared with the 68% of women who had ever been tested for HIV, we found that a larger majority (85%) indicated being likely to take an HIV test if offered. Risk factors and willingness to get tested did not differ significantly between those who had and had not been tested previously, indicating that testing disparities are instead likely caused by barriers to access. Though women may be amenable to HIV testing, few may seek out voluntary testing due to barriers such as stigma and concerns about the lack of anonymity, especially on smaller islands in the Caribbean [16]. These findings echo reports from other developing countries, where researchers have found high interest in getting tested for HIV despite lower uptake [35, 36].

HIV testing is a cornerstone of prevention and a critical component of the HIV care continuum along with subsequent linkage to care and treatment. In the DR, as in many other low-income countries, residents may have access to local public health clinics which serve as the basis for healthcare in a community. The local public health clinics serving these communities are generally within walking distance. Complicated cases presenting to the clinic are referred to the local hospital or the tertiary care centers in surrounding urban centers. Most women in our study had access to medical care and had visited a clinic in the last year. Thus, although barriers to care such as transportation and cost were reported, the engagement of many women with the healthcare system was evident. Despite this, only 68% had ever been tested for HIV, and even fewer (28%) had been tested for other STIs, suggesting that routine medical providers should increase efforts to discuss and offer HIV and STI testing. The major barriers to this practice may be stigma and cultural norms, as reported among healthcare providers in other Hispanic/Latino countries [37]. The goal for this region should be to not only increase access to HIV testing, but also to decrease barriers for women seeking comprehensive STI screening and general health care.

Our study was subject to several limitations. The small sample size may limit the power of the study to detect differences between groups and may also limit the generalizability of the results. Given the limited infrastructure within the community, it was sometimes difficult to determine where one dwelling began and one ended. However, best efforts were made to ensure appropriate sampling methodology. Our findings may also be limited by recall bias, self-report, and select item nonresponse. Our lack of significant findings related to several factors, including education, may reflect these limitations. These areas merit future study with an emphasis on reaching this population more effectively. Despite the limitations of our study, which are inherent to any study of this type, these data represent a novel and important contribution to the literature, with important implications for future work.

## Conclusions

Our study is among the first to report sexual health and HIV testing behaviors among women in the rural DR. Our findings indicate ample opportunities for public health interventions to improve access to health care and HIV testing. Incorporating HIV testing into routine health care, especially salient to a population with relatively high engagement in routine medical care, may represent an effective method of promoting HIV prevention and early diagnosis. This strategy is likely responsible in part for Cuba having the lowest HIV prevalence in the Caribbean [16]. The results of our study provide the basis for future community-based HIV testing initiatives in the DR. These data also provide insights and opportunities on potential areas for public health intervention to improve overall sexual health among women in the rural DR.

## Data Availability

Data are available upon request.

## Abbreviations

AIDS: Acquired immunodeficiency syndrome
DR: Dominican Republic
HIV: Human immunodeficiency virus
IUD: Intrauterine device
MSM: Men who have sex with men
STI: Sexually transmitted infection
